# Impact of COVID-19 on Health Economics and Technology of Diabetes Care: Use Cases of Real-Time Continuous Glucose Monitoring to Transform Health Care During a Global Pandemic

**DOI:** 10.1089/dia.2020.0656

**Published:** 2021-03-02

**Authors:** Sandip Garg, Gregory J. Norman

**Affiliations:** ^1^ Western Digital Corp., San Jose, California, USA.; ^2^ Dexcom, Inc., San Diego, California, USA.

**Keywords:** COVID-19, Telehealth, Digital health, Health technology, Continuous glucose monitoring, Health economics

## Abstract

***Background:*** The coronavirus disease 2019 (COVID-19) pandemic has exposed vulnerabilities and placed tremendous financial pressure on nearly all aspects of the U.S. health care system. Diabetes care is an example of the confluence of the pandemic and heightened importance of technology in changing care delivery. It has been estimated the added total direct U.S. medical cost burden due to COVID-19 to range between $160B (20% of the population infected) and $650B (80% of the population infected) over the course of the pandemic. The corresponding range for the population with diabetes is between $16B and $65B, representing between 5% and 20% of overall diabetes expenditure in the United States. We examine the evidence to support allocating part of this added spend to infrastructure capabilities to accelerate remote monitoring and management of diabetes.

***Methods and Results:*** We reviewed recent topical literature and COVID-19–related analyses in the public health, health technology, and health economics fields in addition to databases and surveys from government sources and the private sector. We summarized findings on use cases for real-time continuous glucose monitoring in the community, for telehealth, and in the hospital setting to highlight the successes and challenges of accelerating the adoption of a digital technology out of necessity during the pandemic and beyond.

***Conclusions:*** One critical and lasting consequence of the pandemic will be the accelerated adoption of digital technology in health care delivery. We conclude by discussing ways in which the changes wrought by COVID-19 from a health care, policy, and economics perspective can add value and are likely to endure postpandemic.

## The Transformative Effects of COVID-19 on U.S. Health Care System and Delivery

### Health care system vulnerabilities despite significant investment

Much like its medical presentation as a virus attacking a vulnerable host, coronavirus disease 2019 (COVID-19) has similarly impacted global health care systems from a health economics and public affairs perspective, both wreaking havoc in certain respects and creating opportunities in others. Only 5% of countries had sufficient funding for epidemic preparedness and <50% had shown they owned supply stockpiles or had agreements with other countries to meet demand surges in a crisis situation.^1^ In the United States, despite health care spending representing ∼20% of 2020 forecasted gross domestic product (GPD), twice that of the global average,^2,3^ the pandemic has exposed vulnerabilities and placed tremendous financial pressure on virtually all nodes of the delivery network. Hospital revenues have declined, in part due to cancellation of surgeries that can account for >50% of the revenue base, supply chains have been strained, and labor costs and intensity have escalated simultaneously to meet new exigent demand.^4^ All of these dynamics are taking place within a hospital system in which nearly a third of institutions have negative operating margins,^5^ underscoring the need for government support, as evidenced by the CARES Act providing $100 billion in emergency funding. America has sadly achieved the undesirable twin designation as the world's number one spender of health care as percentage of GDP and number one in overall COVID-19 cases and deaths.

How did this happen? Part of the underperformance in public health arises from the fact that an estimated 25% of aggregate health care spending in the United States is wasted,^6^ an alarmingly high number compared with other sectors of the economy, depressing health care return on investment (ROI), and its economic viability and sustainability—critical cushions during periods of significant stress. Moreover, the pandemic has exposed interconnected crises in health insurance, financial losses for providers, racial and ethnic disparities, and overall public health infrastructure.^7^ In the earlier stages of the pandemic, estimates of the added direct medical cost burden due to COVID-19 ranged from ∼$160B (20% of the population infected) to ∼$650B (80% of the population infected) over the course of the entire pandemic, with a cost estimate of a single course of infection totaling ∼$3K.^8^ Given ∼10% of the U.S. population has diabetes,^9^ we could impute an incremental spend between $16B (20% infected) and $65B (80% infected) attributable just to this population, which represents between 5% and 20%, respectively, of overall diabetes expenditure.^10^ If we assume that 80% of the population will become infected as a worst-case scenario and that is more representative of estimates of the threshold for herd immunity given current transmission rates, can we then allocate the incremental 20% of diabetes-specific spending to achieve outcomes above and beyond saving lives and avoiding waste, thereby creating opportunity (i.e., ROI) in the midst of crisis? We believe this to be a central question writ large for policy makers and the overall health care system.

This question also looms large when considering socioeconomic and racial/ethnic disparities exposed by the pandemic. The “social determinants of health care” are not new phenomena and are multifaceted. For example, a recently published article by Lipman et al. analyzed the accessibility of new technologies including continuous glucose monitoring (CGM) and pumps by the pediatric type 1 diabetes (T1D) population, finding that racial disparities in technology use and diabetes outcomes (higher hemoglobin A1C [HbA1c], hospitalizations, emergency department visits) persist in children with T1D, regardless of insurance status.^11^ The pandemic has exacerbated these inequities, not only by disproportionately affecting certain populations to the virus itself, but also through the second- and third-order effects of public policies and nonpharmaceutical interventions. For example, social distancing, isolation, travel restrictions, and school closures have impacted household incomes and led to job losses across various industries, especially hospitality, tourism, and manufacturing.^12^

### The role of the pandemic in auguring a new “digital revolution” in health care

Despite the sobering news and performance metrics in the United States, COVID-19 has catalyzed an unprecedented surge in scientific and technology resources, marshalling a type of “all-hands” industrial effort reminiscent of previous global calamities. Indeed, the disease has spawned the fastest scientific response in history from an R&D perspective—nearly 3K research articles were published within the first 3 months of the outbreak alone.^13^ But perhaps one of the most lasting consequences will be the (forced) accelerated adoption of technology in health care delivery. The digital health care market is estimated to be >$100B, growing at ∼30% compound annual growth rate between 2020 and 2025,^14^ but the adoption and trust of new technologies in health care have been challenging despite its secular growth. In a 2019 analysis by PricewaterhouseCoopers, ∼40% of heads of major U.S. health care systems reported having no digital component in their overall strategic plan, with >90% of these respondents blaming data protection and privacy issues as hindrances.^15^ Physician burnout has been another major headwind for technology adoption. And financial incentives have long favored incumbent ways of doing business—consider, for example, that a typical hospital makes 10–20 × more if a patient visits the emergency room versus using an online platform to talk with a telehealth provider.^14^

Demand has surged for alternatives to in-person health care delivery during the COVID-19 pandemic. In the earlier days of the pandemic, congress authorized telemedicine services for all beneficiaries of fee-for-service Medicare as an initial step, but wider adoption is contingent on aligning on reimbursement protocols, updating regulations, and evaluating clinical care provided by such technologies.^15^ Nonetheless, the results have been impressive: telemedicine use has surged from pre-40% penetration to >60%, large employers were citing 30%–40% utilization rates in April and industry publications have cited a 30 × increase in broader telemedicine utilization, and the Teledoc/Livongo merger was consummated as a poster child of the new digital revolution.^16^ The health care sector and capital markets are clearly favoring companies and organizations using technology not just as an enabling platform, but also as a central driver of business value. As a result, management teams and leaders are adjusting in real time: ∼60% of health care industry leaders say that flexible/hybrid office–home work will become a staple; 51% say there will be greater focus on automation, technology, and data analytics; and 75% say there will be significantly greater use of virtual health care delivery.^17^

Diabetes care is a superb example of the confluence of the pandemic and heightened importance of technology in changing care delivery and maximizing outcomes despite macro challenges. Diabetes has been established as a risk factor for a poor prognosis for COVID-19.^18–20^ In a retrospective study of patients with COVID-19 in China, Zhu et al.^21^ found a strong association between glucose control and outcomes in patients with COVID-19 and pre-existing type 2 diabetes (T2D). Lockdown and social distancing during the pandemic have had worldwide negative economic impacts with ramifications for health care access and delivery.

One technology that has shown to be helpful during the pandemic is real-time continuous glucose monitoring (rtCGM). Numerous studies have shown rtCGM helps people with diabetes improve and manage glycemic control for reducing both hyperglycemia and hypoglycemia.^22–25^ Hereunder we highlight three areas wherein rtCGM has been an effective tool for managing diabetes during the COVID-19 pandemic. First, we look at evidence suggesting that rtCGM helps individuals in the community manage their diabetes during lockdown; second we consider how rtCGM is being used for telehealth to help clinics remotely manage their patients; and third, we summarize how rtCGM is being used in hospitals to monitor patient glucose levels and limit health care provider exposure to COVID-19.

## Use Cases of rtCGM During the COVID-19 Pandemic

### rtCGM use in the community during lockdown due to COVID-19

During the first months of the pandemic, governments around the world implemented “lockdown” restrictions in an effort to minimize the spread of the virus. People were advised to stay at home and avoid all unnecessary travel outside the home to essential activities such as purchasing food. Although such restrictions impacted the lives of all individuals, people with chronic diseases such as diabetes could have been seriously impacted by restrictions that changed routine diet and physical activity patterns, increased stress and anxiety, and reduced access to health care resources.^26,27^ Maintaining glycemic control can be affected by all of these factors.

Interestingly, several recently published studies indicate people with T1D using CGM generally had improved time in range (TIR) and reduced glucose variability.^26–30^ For example, Brener et al. found CGM metrics in pediatric T1D patients were stable during lockdown in Israel.^28^ The same pattern of improved glycemic control for adults with T1D using CGM was found in Spain,^26^ Italy,^27,29^ and the United Kingdom.^30,31^ Van der Linden (in this issue) compared rtCGM Dexcom G6 data uploaded by >60,000 individual patients before and during the pandemic and found TIR improved on average 2.3% from prepandemic to the early months of the pandemic (March through June 2020). The pandemic-related improvements in TIR were greater in areas with higher median incomes, highlighting one of the consequences of wealth inequality in the U.S. areas with higher COVID-19 burden was associated with greater improvements in TIR, suggesting increased attentiveness to diabetes management in areas where risk of infection was higher.^32^

Lockdown during the early months of the pandemic may have made some aspects of diabetes management easier for some people through changes such as eating at home instead of eating at restaurants, having more stable schedules, and having more time for self-management may help to improve glycemic control. It may also be the case that increased public awareness that diabetes is a risk factor for poor prognosis of COVID-19 may motivate people with diabetes to more carefully manage their glycemic control.^26^ However, it is not clear whether this pattern would continue over a longer period of lockdown.

Other U.S. data indicate young (age 0–24 years) T1D patients with confirmed COVID-19 were more likely to be on public insurance, have a higher HbA1c, and not using CGM or an insulin pump if they were non-Hispanic black or Hispanic compared with non-Hispanic white patients.^33^ Ebekozien et al. also found non-Hispanic black and Hispanic patients with COVID-19 were more likely to be hospitalized and more likely to present with diabetic ketoacidosis (DKA) than non-Hispanic white patients.^33^ Higher levels of socioeconomic deprivation were associated with deteriorating glycemic control during lockdown in the United Kingdom.^30^ Similarly, younger Israeli children with T1D in lower socioeconomic areas had higher glucose levels during lockdown. The lockdown in response to the pandemic seems to exacerbate the socioeconomic crisis for those already in financial difficulty.^12,34^

rtCGM can clearly serve as a surveillance tool to monitor population trends in glycemic control during public health crises. The use of rtCGM may also have a protective effect on patients with diabetes to help mitigate the severity of COVID-19 by helping to optimize glycemic control. Unfortunately, less is known about trends in diabetes management among those not using CGM.

### rtCGM for telehealth for remote patient management

There has been increased interest in telehealth from patients and providers with a number of studies demonstrating feasibility and improved clinical outcomes of telemedicine interventions for diabetes.^35–37^ Telemedicine during the pandemic provides a means to conduct patient care while minimizing the risk of exposure and transmission of the virus.^38^ Garg et al. presented two case studies of new-onset T1D management during the pandemic.^39^ In one case of an adult male, Dexcom G6 rtCGM and CLARITY software were used to facilitate virtual care. Insulin dose was adjusted daily for the first 7 days by the physician based on CLARITY summary reports. During the second week, a certified diabetes educator conducted televisits. The second case study was of a 12-month old, who upon diagnosis was started on CGM and an insulin pump. A similar approach to using CLARITY was taken to remotely monitor glucose and titrate insulin during the first 2 weeks after diagnosis. Both case studies show that digital remote care is feasible and acceptable to patients and providers, even for difficult situations such as new onset T1D during the pandemic. In addition, the role of rtCGM in managing diabetes during pregnancy, managing DKA during the early months of the lockdown, and as part of a virtual clinic for managing T2D have also been described.^40–42^

In the United States, the pandemic has resulted in the removal of some longstanding regulatory burdens to telehealth. Health and Human Services (HHS) has made it easier to provide care through telehealth during the COVID-19 pandemic, including use of common communication apps (e.g., FaceTime [Apple, Inc.] and Zoom [Zoom Video Communications, Inc.]) as well as waivers to conduct telehealth to patients in homes outside of rural areas, provide telehealth care across state lines, televisits to new and established patients, and to bill for telehealth as if care were provided in person.^43^

CLARITY clinic software provides clinics with a web-based portal to view their patients' CGM summary data. Norman et al. found evidence CLARITY use increased at clinics serving patients with diabetes during the pandemic.^44^ They examined use of CLARITY by clinics in the United States from January to July 2020 and compared monthly year-over-year (YOY) changes with 2019. It was hypothesized that an increase in clinic staff logins to CLARITY after March 2020 would indicate that health care providers were using CGM for remote telehealth during the pandemic.

Although the monthly number of new clinic registrations to CLARITY was not related to the timing of the pandemic, the monthly number of clinic logins to CLARITY from March to April significantly increased in 2020 compared with the same period in 2019. This increase in logins started in April and continued through July. For example, in 2020, the total monthly clinic logins increased from 71,012 to 95,088, which was a 34% increase from January to July. Compared with 2019, the YOY increase in monthly total logins was between 49% for January and 99% for June. Between April and July, the YOY change in logins was higher than expected with at least 70% YOY increase each month. These data suggest that clinics increased their use of CLARITY to manage their patients with diabetes in response to the pandemic.

### Use of rtCGM in place of point-of-care testing for glucose management in the hospital

rtCGM is currently not Food and Drug Administration (FDA) approved in the United States for inpatient hospital use, and point-of-care (POC) testing has been the standard of care for glucose management. There has been ongoing expert discussion on uses of CGM in the inpatient setting for both intensive care unit (ICU) and non-ICU.^45,46^ Use of older CGM models in the hospital setting was prohibited because of barriers such as poor accuracy, need for calibration, interference from acetaminophen use, sensor drift, and measurement lag.^45^ However, newer rtCGM devices, such as the Dexcom G6, have eliminated these barriers and achieve accuracy levels below a mean absolute relative difference (MARD) of 10%.^47^ Recent studies where non-ICU patients were randomized to rtCGM or POC testing showed rtCGM resulted in improved glycemic management for patients with T2D,^48^ and decreased hypoglycemia for high-risk insulin-treated patients with T2D.^49^

Because diabetes is associated with increased risk for morbidity and mortality for patients in the hospital infected with COVID-19,^20,50^ the pandemic has created an urgency to determine the feasibility of CGM in the hospital.^51,52^ On April 1, 2020, the FDA exercised “enforcement discretion” of CGM use in hospitals because they determined the risk to patients was low. This decision allowed hospitals to use CGM for remote monitoring of patients to reduce personal protective equipment use and limit health care workers' exposure to the virus.^53^

Several recent publications report on inpatient hospital use of CGM during the COVID-19 pandemic. An earlier pilot study tested the feasibility of CGM in the hospital for noncritically ill COVID-19 patients and found high accuracy and reduced POC testing.^54^ Shehav-Zaltzman et al. described the use of CGM in two hospital isolation wards where monitoring stations were created using CGM systems.^55^ They found CGM for remote real-time diabetes management feasible and added to the quality of care while minimizing health care work exposure risk. A case report on a severely ill patient with diabetes and pneumonia caused by COVID-19 demonstrated successful use of Dexcom G4 rtCGM for remote monitoring of the patient to track glucose levels from outside the patient's isolation room.^56^ These studies emphasized the added infrastructure needs for safe implementation of CGM in the hospital, including staff education, establishing protocols for device placement and replacement, monitoring device accuracy, and integrating CGM readings into the medical record.^57^

## Perspectives on the Future, Post-COVID-19

Heeding Churchill's famous admonition for future generations to “never let a good crisis go to waste,” we conclude by discussing ways in which the changes wrought by COVID-19 from a health care, policy, and economics perspective are likely to endure long after the pandemic subsides. If the operative question for society is how to achieve meaningful ROI in the midst of crisis, to see that the incremental 20% health care spend attributable to COVID-19 and diabetes leads to sustainable change that makes medical practice more efficient and equitable, then we believe the winner in this whole experience will be the accelerated use and adoption of technology as the *de facto* “new normal” post-COVID-19.

First, from a health care and policy perspective, clearly the twin rise of telehealth services in medicine broadly and the use of rtCGM in the diabetes market specifically as discussed in this article have demonstrated their value proposition to providers, patients, payers, and regulators. All of the R&D investment that such technologies are leveraging has been put to the test in the most exigent of circumstances and shown safety and efficacy across nearly every performance indicator.

Although industry and the clinic seem to have moved forward in technology adoption, as well as the overall marketplace, regulators and policy makers will need to carefully articulate the appropriate guardrails and safeguards for such technologies to ensure compliance, mitigate abuse, maintain safety standards, and encourage future innovation. This approach would ensure that today's inflection point in technology will not be undercut by intractable privacy or security concerns and would rather continue the journey toward a more technology-enabled and efficient future in health care delivery and practice.

Moreover, the pandemic has acutely highlighted health care disparities based on socioeconomic factors, an area where technology could have a significant positive impact if deployed strategically and with appropriate regulatory backing.^58^ For example, CMS has loosened requirements for obtaining CGM during the COVID-19 pandemic such as not having to go to the doctor's office to receive CGM and not having to demonstrate use of four finger sticks a day, a requirement for CGM eligibility. Making these regulatory changes permanent will help increase access to CGM for those who can benefit from the technology. In addition, even though, at present, 35 states provide some coverage for CGM through Medicaid, expanding Medicaid coverage for CGM to all states for patients taking insulin for diabetes will help to close socioeconomic disparity gaps for those in the United States without private health insurance. Beyond policy change for reimbursement of health technologies such as CGM, disparities in access to health technologies continue to exist when access to diabetes specialists and diabetes educators is limited in areas of socioeconomic deprivation.^59^

Second, by virtue of their forward-looking orientation, the capital markets and investment community have already moved forward in assigning winners and losers in the post-COVID-19 world. The winners are indisputably companies and organizations that place technology at the center of what they do—consider that over the past year, at the time of this writing, the S&P Technology Index is up 44% while the S&P 500 overall index is up 18%, and that all of the equity market growth this year has been driven by large-cap technology companies.^60^ Stock markets are forward-looking barometers of risk and help reveal the market consensus on future trends. As another example of what the post-COVID-19 world will look like, Salesforce announced on 12/1 its acquisition of Slack Technologies, a business communications and virtual workload software tool, for $28 billion.^61^ That this transaction would be announced 9 months into a global pandemic and would represent the largest ever acquisition by Salesforce, a $200B market cap global technology leader, underscores the gravity of the secular change envisioned in technology use in the new world post-COVID-19.

As we have discussed, these changes are also bearing fruit in the health care world, where the growth in the reliance on technology to provide care during the pandemic is changing incumbent ways of doing business and will likely lead to positive ROI. At the Cleveland Clinic, for example, the population health analytics team is using algorithms proactively to reduce COVID-19 hospitalizations by 7.5%, geomapping COVID-19 exposures to determine hotspots before even health officials have such data on their radar, and using technology to enable value-based work at scale.^62^ Similarly, we have highlighted successful use cases of rtCGM during the pandemic to manage diabetes safely and effectively, which can continue to add value post-COVID-19. We envision more such success stories across the health care landscape and in diabetes especially. With the right set of policies and infrastructure in place, COVID-19 may transform the health care system to better serve the broader public's needs in an economically efficient way over the next 100 years.
